# Evidence-based advice on timing and location of tsetse control measures in Shimba Hills National reserve, Kenya

**DOI:** 10.1371/journal.pntd.0011398

**Published:** 2023-06-05

**Authors:** Stella Gachoki, Thomas A. Groen, Anton Vrieling, Andrew Skidmore, Daniel Masiga

**Affiliations:** 1 International Centre of Insect Physiology and Ecology (*icipe*), Nairobi, Kenya; 2 University of Twente, Faculty of Geo-Information Science and Earth Observation (ITC), Enschede, the Netherlands; Foundation for Innovative New Diagnostics (FIND), SWITZERLAND

## Abstract

Controlling tsetse flies is critical for effective management of African trypanosomiasis in Sub-Saharan Africa. To enhance timely and targeted deployment of tsetse control strategies a better understanding of their temporal dynamics is paramount. A few empirical studies have explained and predicted tsetse numbers across space and time, but the resulting models may not easily scale to other areas. We used tsetse catches from 160 traps monitored between 2017 and 2019 around Shimba Hills National Reserve in Kenya, a known tsetse and trypanosomiasis hotspot. Traps were divided into two groups: proximal (<1.0 km)) to and distant (> 1.0 km) from the outer edge of the reserve boundary. We fitted zero-inflated Poisson and generalized linear regression models for each group using as temporal predictors rainfall, NDVI (Normalized Difference Vegetation Index), and LST (land surface temperature). For each predictor, we assessed their relationship with tsetse abundance using time lags from 10 days up to 60 days before the last tsetse collection date of each trap. Tsetse numbers decreased as distance from the outside of reserve increased. Proximity to croplands, grasslands, woodlands, and the reserve boundary were the key predictors for proximal traps. Tsetse numbers rose after a month of increased rainfall and the following increase in NDVI values but started to decline if the rains persisted beyond a month for distant traps. Specifically, tsetse flies were more abundant in areas with NDVI values greater than 0.7 for the distant group. The study suggests that tsetse control efforts beyond 1.0 km of the reserve boundary should be implemented after a month of increased rains in areas having NDVI values greater than 0.7. To manage tsetse flies effectively within a 1.0 km radius of the reserve boundary, continuous measures such as establishing an insecticide-treated trap or target barrier around the reserve boundary are needed.

## Author summary

To control African trypanosomiasis, tsetse populations must be reduced to levels that impede disease transmission. Limited reliable information on tsetse temporal dynamics hinders the implementation of effective control strategies. We used two empirical modelling strategies (zero-inflated Poisson and generalized linear Poisson) to link tsetse abundance with environmental and weather data, to understand and explain tsetse temporal dynamics in a heterogeneous environment. Tsetse numbers were consistently high within 1.0 km of the reserve outer boundary due to favorable breeding sites, resting sites, and host availability. Beyond 1.0 km of the reserve boundary, tsetse numbers increased after a month of increased rainfall, but started to decline if rainfall persisted beyond a month. For these localities, higher tsetse numbers were also found in areas with NDVI values above 0.7. These insights provide valuable guidance for the timing and location of tsetse control strategies around Shimba Hills National Reserve, particularly considering the limited resources available.

## Introduction

Tsetse flies are the sole biological vector of both human and animal African Trypanosomiasis (AT) in 38 Sub-Saharan African (SSA) countries at locations where its suitable habitats are predicted to occur [1,2]. The interaction between tsetse flies, wild and domesticated hosts, and the trypanosome pathogens determines the epidemiology of cyclic trypanosomiasis. Although vaccines do not exist for either of the diseases, the number of human AT cases have fallen significantly in the last decade. Recently, the *T*. *b*. *gambiense* form of human AT has been eliminated in Benin, Uganda and Rwanda [3]. However, progress on animal AT control is much slower [4]. Chemotherapy and the use of trypanocide drugs are widely used to control trypanosomiasis in infected livestock [5], but the emergence of drug-resistant trypanosome pathogens has rendered current drugs less effective [5,6], with over three million cattle heads dying each year globally [7–10]. As a result, the most effective way to control the disease is to reduce tsetse numbers to a level that decreases or inhibits disease transmission [11].

Several factors are known to influence tsetse abundance. Temperature is one of the major factors controlling the physiological processes of tsetse flies [12,13] and thus most of its population dynamics. Increases in temperature increase adult mortality rates, which lowers the population, while at the same time increasing tsetse reproduction rate and declining the time taken for pupae to emerge [13,14] increasing the population. On the other hand, low body temperatures of tsetse are likely to induce chill coma in adult flies [15], lengthen the time taken for eggs to develop in the female ovaries as well as the time taken for pupae to emerge [13,16,17], lowering populations. In Zimbabwe increases in temperature over time have been linked to a significant decrease in tsetse fly numbers [11,12], and increase in their reproductive rates [17]. However, as temperatures continue to rise, the reproductive rates of tsetse flies begin to decline due to increased pupal mortality rates [17]. Although there is no known direct relationship between rainfall and tsetse fly numbers, moist conditions are essential for burrowed tsetse pupae to emerge [18]. Nevertheless, periods of heavy rainfall can have detrimental effects on tsetse numbers. For example, a) when flooding occurs, pupae (6-7mm length) buried in loose soil may be washed away; b) rainfall periods could lower mean temperatures thereby reducing female reproduction rates or c) high humidity levels can saturate the atmosphere and cause low evaporation rates which could result in increased soil moisture reducing the survival rate of larvae [19,20]. Variations in rainfall can also explain changes in the Normalized Difference Vegetation Index (NDVI—a measure of green biomass; [21,22]) which has been widely used to explain the spatial presence of tsetse flies [23–26]. Green vegetation is likely to provide better shaded and cool conditions for tsetse flies to rest and breed, but no studies have been conducted that use NDVI to explain temporal variation in tsetse densities.

In Kenya, there is a positive correlation between the occurrence of tsetse flies and the locations of protected areas (Kenya Tsetse and Trypanosomiasis Eradication Council). Although protected areas serve as breeding hotspots for tsetse flies, these flies are found to be distributed extensively beyond the boundaries of these protected areas [27,28]. The Kenya Wildlife Service (KWS) manages these protected areas and prohibits the control of tsetse flies within them, as part of its duty to protect all animals, thereby preventing the deployment of odour-baited targets at any density within the national reserve. In Kenya, *Glossina pallidipes* is the most common and widely spread tsetse species [29] making it the most significant in the transmission of animal trypanosomiasis. Despite its significance, information on its abundance is limited to a few locations that have been targeted for monitoring. Even in areas where these data are available, there is still a lack of understanding of how tsetse numbers change over time and the role that environmental and weather variability play. This could be because the adaptability of *G*. *pallidipes* under diverse environmental conditions [30], poses a challenge in pinpointing the primary environmental factors that govern its dynamics in natural habitat. Given the difficulty for collecting *in-situ* data on tsetse abundance for large areas and longer time frames, satellite data can be a useful tool to predict densities by deriving environmental and weather variables that influence the development and behaviour of disease vectors such as tsetse flies. The purpose of this study was to determine if satellite-derived environmental factors and weather data can be used to explain the abundance of *G*. *pallidipes* around the Shimba Hills National Reserve. Specifically, we aimed to 1) analyze where and when *G*. *pallidipes* numbers were high based on trapping data; 2) assess the environmental and weather conditions that may explain the observed temporal abundance dynamics; and 3) use the evidence obtained to discuss a management strategy for tsetse control.

## Data and methods

### Study area

**Fig 1:**
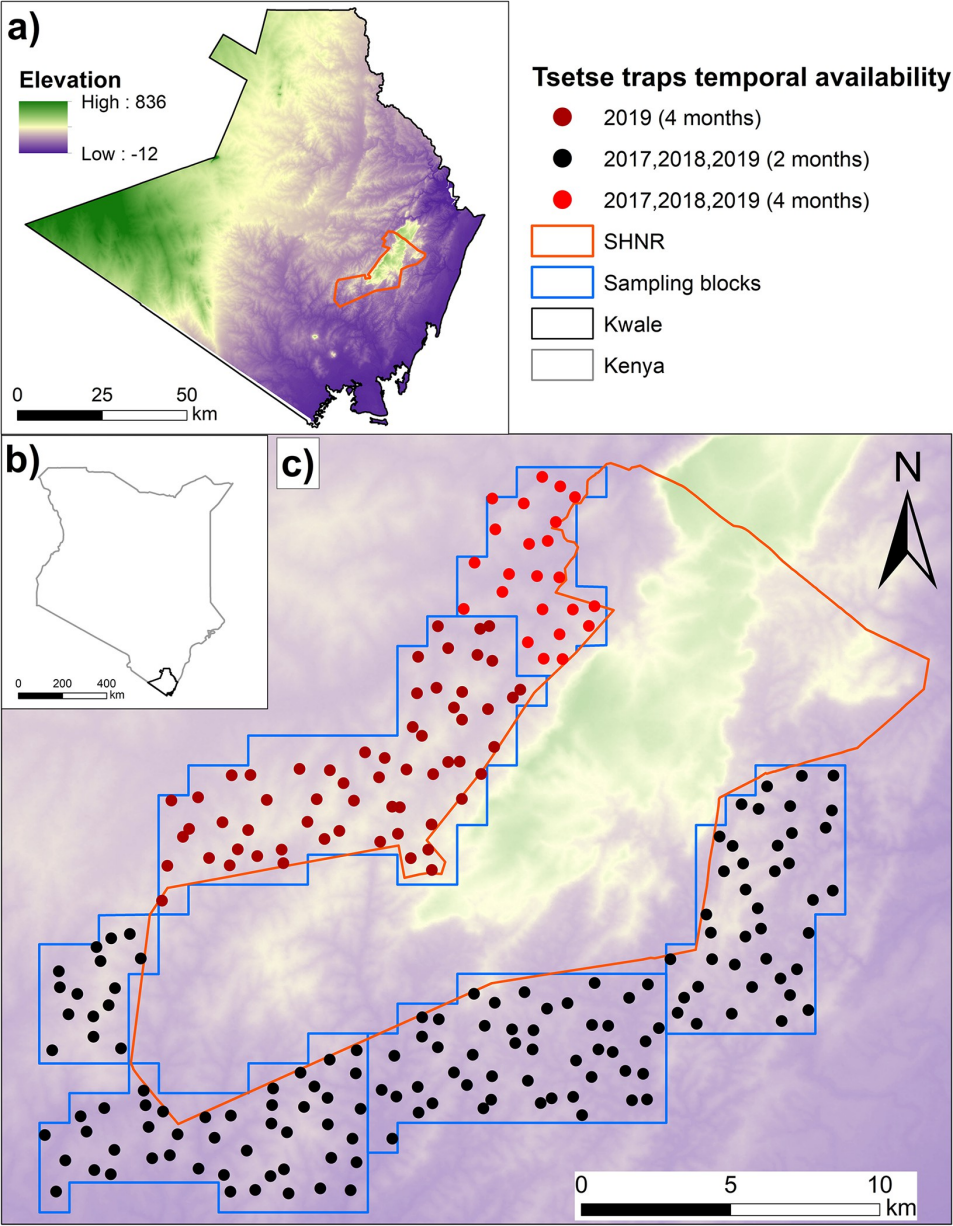
Location of the study area. a) Kwale county boundary, the background shows the 30m-resolution digital elevation model from the Shuttle Radar Topography Mission (SRTM) as provided by United States Geological Survey (USGS; https://cmr.earthdata.nasa.gov/search/concepts/C1000000240-LPDAAC_ECS.html). b) Kwale county location within Kenya (source; https://africaopendata.org/dataset/kenya-counties-shapefile). c) Shimba Hills National Reserve (source; https://geoportal.icpac.net/layers/geonode:ken_protected_areas), the dots are the tsetse trapping locations and are color-coded based on the period they were monitored.

Our study area was the area surrounding Shimba Hills National Reserve (SHNR; 235 km^2^) in Kwale County of Kenya (**Fig 1**). The reserve is one of Kenya’s 65 protected areas, with a hot and humid climate and annual rainfall ranging from 900 to 1500 mm. SHNR is covered with patches of natural forest, dense thickets, and grasslands with scattered shrubs. The reserve also serves as a haven for a diverse range of wild animals, including warthogs (*Phacochoerus africanus*) and bush pigs (*Potamochoerus porcus*), which are among the most preferred hosts for *G*. *pallidipes* to feed on [31]. Farmers that cultivate crops and keep livestock (cattle, goats, chicken, etc) populate the communities surrounding SHNR. The main crops for subsistence farming include maize and cassava, while the main cash crops are coconuts, mangoes, oranges, and cashew nuts. Frequent encounters occur between livestock and wildlife near the reserve, increasing the transmission of trypanosomiasis from wildlife (trypanosomes reservoirs) to livestock [32]. As a result, animal AT continues to be a significant constraint to cattle production in the SHNR area despite the continued efforts of control programs [27,33].

### Tsetse fly count data

Between 2017 and 2019, 230 biconical traps that were baited with cow urine and acetone were used to monitor tsetse flies. These traps were deployed at random locations within a 1km grid, extending up to 5km from the reserve boundary. The number of traps monitored varied across different months (**Table 1** and **Fig 1** coloured dots). A total of 9,060 tsetse flies were captured during this period, with *G*. *pallidipes* comprising 98% of the catch. The remaining flies belonged to *G*. *austeni* and *G*. *brevipalpis* but were only collected in five traps. In every period that the data were collected, traps were emptied every two days for four repeats. At the end of the fourth collection, the traps were removed and installed again during the next field campaign. Out of a total of 230 monitored traps, 70 traps were removed from further analysis as they did not capture any flies during the monitoring period. For this study, for each trap, data that were collected within the same period were combined and are referred to as a single collection. Since all traps were monitored for the same period (8 days) there was no need for further standardization.

**Table 1:** The temporal availability of tsetse count data in Shimba Hills.

Trap setting date	Last Collection	Year	No. of traps	Total catches
27-May	03-Jun	2017	126	166
20-Jul	27-Jul	2017	126	144
26-Aug	02-Sep	2017	126	137
18-Oct	23-Oct	2017	126	302
21-Nov	28-Nov	2017	126	984
02-Feb	09-Feb	2018	126	285
07-Mar	14-Mar	2018	126	342
11-Apr	18-Apr	2018	126	435
30-Apr	07-May	2018	126	405
15-Jun	22-Jun	2018	126	206
08-Jul	15-Jul	2018	126	181
20-Feb	27-Feb	2019	55	1056
11-Apr	18-Apr	2019	55	244
21-Jun	28-Jun	2019	160	2130
01-Sep	08-Sep	2019	160	1923

**Fig 2:**
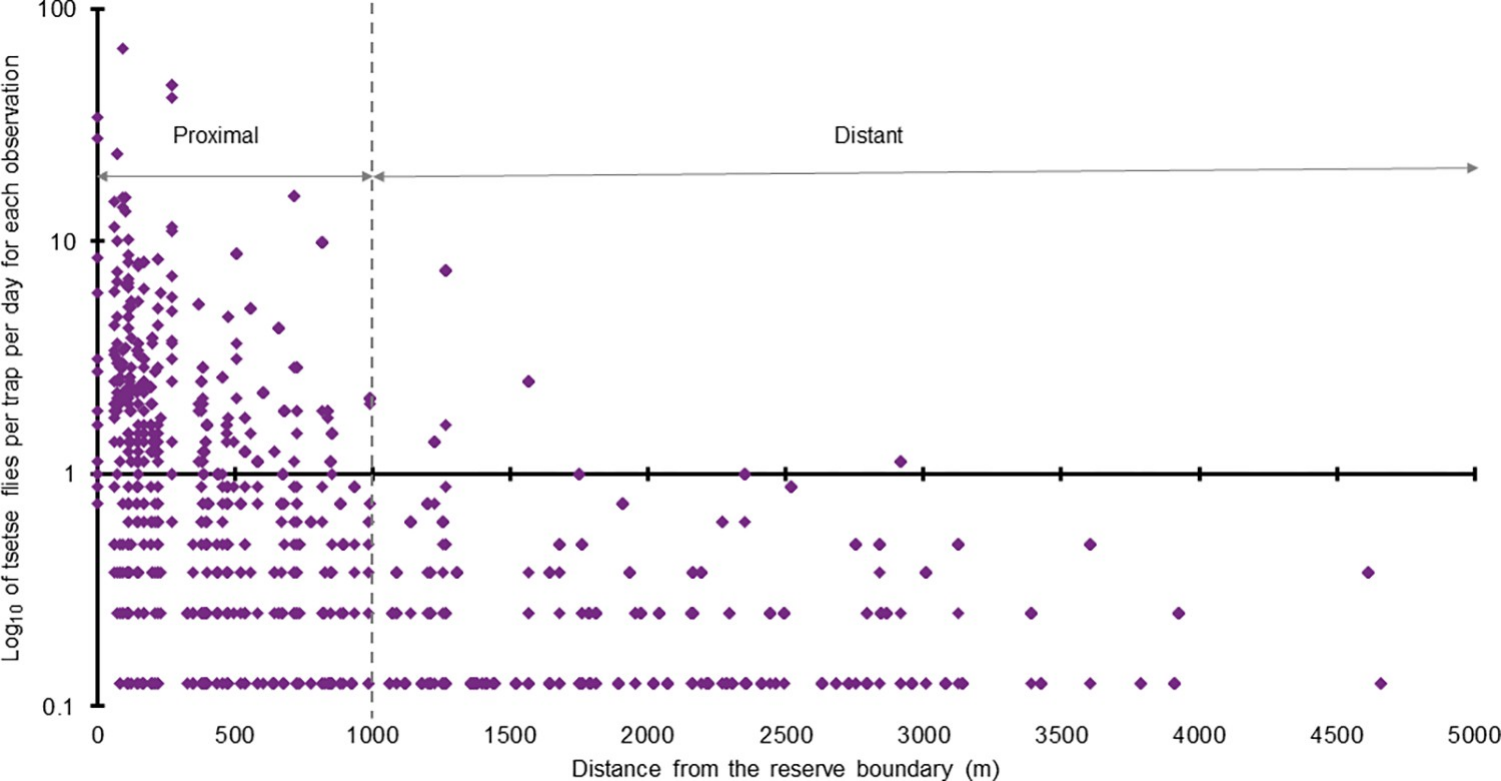
The natural logarithmic scale (base 10) of the number of G. pallidipes per trap per day for each observation (i.e., trap per period) plotted against their distance from the reserve boundary. The dotted line shows the threshold distance from the reserve outer boundary used to group the proximal (<1.0 km) and the distant traps (>1.0 km).

Initial analysis indicated a decrease in tsetse numbers with increasing distance from the outside of the reserve boundary, with most captures occurring within 1km of the boundary (**Fig 2**). To further investigate this trend, we divided the trapping locations into two groups: <1.0 km (proximal group) and >1.0 km (distant group). Each group was linked to the various environmental datasets separately.

### Environmental and climatic variables

Tsetse fly population density is influenced by host availability, temperature, and moisture [34,35]. Spatial data for host abundance is hard to obtain, and while other factors like temperature and moisture can be monitored using satellite sensors, optical sensors are constrained by cloud cover. Moreover, finer spatial resolution observations are generally made less frequent, which affects their timelines and availability throughout the season. We focused on freely available environmental and weather factors that did not require pre-processing. These factors included 10-day NDVI composites produced by BOKU (University of Natural Resources and Life Sciences, Vienna) for the JRC-ASAP (Joint Research Centre–Anomaly Hotspots of Agricultural Production) at 1km, derived 10-day Land Surface Temperature (LST) composites provided by Meteosat Third Generation at ~5km and daily Climate Hazards Group InfraRed Precipitation with Station data (CHIRPS) data with a spatial resolution of 5km. For NDVI and LST, we used composites that covered 60 days before the last day of tsetse collection for each trap in steps of 10 days and a total of six composites (t1-t6, whereby t1 was 1–10 days before tsetse collection, t2 was 11–20 days, t3 was 21–30 days, t4 was 31–40 days, t5 was 41–50 days, and t6 was 51–60 days). For the daily CHIRPS data, we generated 10-daily sums (i.e., total rainfall within the 10 days for every pixel) also going back to 60 days before the last date that tsetse was monitored. For readability, precipitation was annotated with P, and the time lag variables were renamed as P_t1_ to P_t6_. Besides the temporal-varying variables, we also used the distance of each trap to the reserve boundary, and to the nearest woodland, cropland, and grassland land cover (D_park_, D_wood_, D_crop_, and D_grass_, respectively) to help explain spatial variability in tsetse abundance. **Table 2** details the predictor variables used and the rationale for including them.

**Table 2:** Environmental variables used to relate with tsetse fly numbers.

Data	Spatial resolution (m)	Temporal resolution (days)	Source/reference	Hypothesis
NDVI	1000	10	MODIS	We hypothesize that tsetse abundance will increase as NDVI values rise. This is because high NDVI values indicate high vegetation greenness, which could indicate the presence of cool shaded areas that encourage tsetse fly breeding [36] and thus population growth.
Precipitation (P)	5000	10	CHIRPS	Tsetse abundance is expected to decrease due to flooding if heavy rainfall occurs close to periods when tsetse was monitored [18], but an increase in rainfall one to two months before tsetse collection will increase abundance [37].
LST	5000	10	LSA SAF	Temperatures above 32°C or below 16°C are expected to result in fewer tsetse flies being trapped [13,38,39].
Distance to the reserve (D_park_)	-	-		Tsetse fly localities in Kenya are positively correlated with protected areas that have abundant wild hosts and shading, so we expected tsetse numbers to decrease when moving away from the reserve.
Distance to woodlands (D_wood_)	-	-	Gachoki *et al*. [24]	Tsetse abundance will decline when moving away from the woodlands because *G*. *pallidipes* prefer woody vegetation that provides sufficient shade for resting and breeding.
Distance to croplands (D_crop_)	-	-	Gachoki *et al*. [24]	Human interference, such as cropping, negatively affects tsetse habitat. Therefore, we expected a higher abundance further from the croplands.
Distance to grasslands (D_grass_)	-	-	Gachoki *et al*. [24]	We hypothesized that for traps closer to the reserve, tsetse numbers would rise near the grasslands because these could be potential grazing zones resulting in host availability, whereas for traps further away, tsetse numbers would decline because these could have been vegetated cropland fields.

Our dataset of predictors comprised all six 10-day composites for NDVI, LST, and rainfall (P); we refer to this dataset as D1. In addition, we generated two alternative predictor datasets:

- D2: we kept the “current conditions” t1, but averaged NDVI_t2-t6_, maximum LST_t2-t6,_ and total P_t2-t6_;
- D3: we averaged NDVI_t1-t3_ and NDVI_t4-t6_, and took maximum LST_t1-t3_ and LST_t4-t6_ and total P _t1-t3_ and P _t4-t6_.

**Fig 3:**
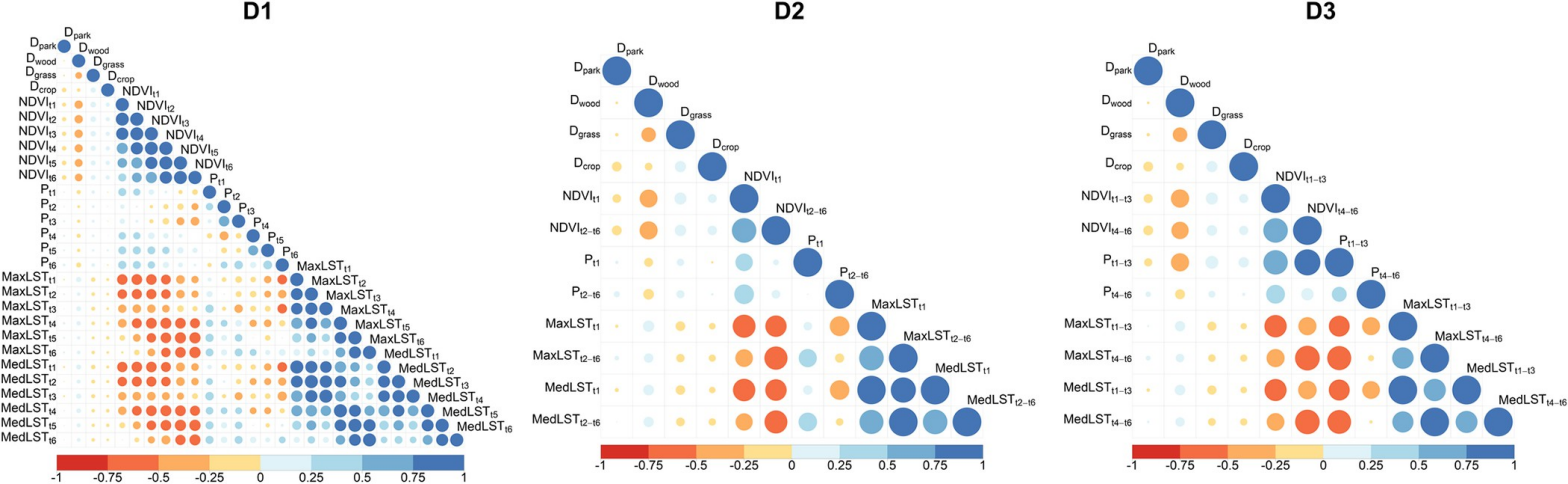
Correlation matrix of the predictor variables for the different datasets (D1 = 10-day variables of t1-t6; D2 = t1 and averaged t2-t6; D3 = averages of t1-t3 and t4-t6). Blue colours represent positive collinearity, while red colours represent negative collinearity. The size of the symbol indicates the strength of the correlation.

To better understand the interaction between the predictor variables, we plotted a correlation matrix for the predictors that were used together in each dataset (**Fig 3**). Most observed correlations were of the same variables at different time lags, which was to be expected. We also found negative correlations between NDVI and LST time lags. Because the strong correlations between NDVI and LST could affect modelling results, we fitted models that included both variables as well as models that excluded LST. We decided to exclude LST, rather than NDVI because it had a lower spatial resolution (5 km) than NDVI (1 km).

### Statistical models

We organized the data such that each record consisted of a single trap monitored during one of the collection periods in **Table 1**. To further clarify; if a trap was monitored in all 15 periods, this resulted in 15 records. In total, we had 1815 observations (n = 1001 for distant and n = 814 for proximal). For each of these records, we extracted the predictor variables described in **Table 2**. Given that the tsetse data was over-dispersed and contained high numbers of zeros (80% in distant group and 34% in proximal group), we tested two modelling techniques to fit empirical relationships: 1) the Zero-inflated Poisson (ZIP) regression model [40], which effectively deals with overdispersion in data and 2) the Generalized Linear regression Modelling (GLM) with Poisson family [41,42], which assumes that the data mean equals the variance. We used the *pscl* package [43] to fit a ZIP model across all datasets (D1, D2, D3) for both groups and the *mpath* package [44] to run a backward stepwise regression, while the Poisson GLM was fitted using both using the *MASS* package [45] in R programming. We started the modelling by fitting Poisson GLM and ZIP models with all the predictor variables, i.e., including the correlated LST and NDVI variables and also without the LST (the coarser-resolution variable of the two) across the three datasets (D1, D2, D3). To assess the models’ performance, we compared the absolute Root Mean Squared Error (aRMSE), relative RMSE (rRMSE; mean/aRMSE), and the McFadden’s pseudo R^2^ for predicted versus observed plots across all models from various datasets. To test the robustness of the identified variables, we also assessed the contribution of variables in explaining variation in tsetse abundance by applying a randomisation procedure. Specifically, we fitted 100 models on a random subset of 700 observations every time a model was fitted, similar to the approach used by Bautista-Cespedes *et al*. [46]. The routine is only available routine single models and does not support “dual model” structure of ZIP model and thus we only applied it to Poisson GLM. Apart from identifying the contributing variables, we determined their importance by calculating the average p-values of the model coefficients from the 100 randomized models: low p-value indicate more significant variable. To determine the relationship between the predictor variables and the tsetse fly count observations, we examined the sign of the fitted regression coefficient.

## Results

### Model performance

The explanatory variables used to predict tsetse numbers in both proximal and distant traps explained only a small portion of the temporal fluctuations in tsetse numbers, which was evident from the low McFadden R^2^ values obtained from the models (**Table 3**). Even so, the randomised GLM modelling strategies exhibited higher R^2^ values compared to ZIP models where R^2^ values obtained using GLM for distant traps varied between 0.16 and 0.37, depending on the dataset used (D1, D2, D3 with (a) or without (b) LST) and 0.34 to 0.53 for proximal traps (**Table 3**). For this modelling strategy, incorporating 10-day time-varying variables with LST (D1 (a)) resulted in lower AIC values (1.14*103). This indicates that this model has a better ability to capture the goodness of fit and complexity when compared to the models that utilized averaged datasets. The aRMSE values exceeded the mean tsetse count in both groups (**Table 3**), implying overestimation by the models. A greater precision of the models was exhibited in proximal traps as the lower rRMSE values (**Table 3**) correspond to lower residual variance.

**Table 3:** Model evaluation statistics for distant and proximal traps across the three modelling strategies. D1 includes t1-t6, D2 includes t1 and averaged t2-t6, and D3 includes averaged t1-t3 and t4-t6. The models labeled with (a) are those with LST and (b) those without LST. The bold figures represent the least rRMSE values, indicating lower residual variance. The AIC values are divided by 1000.

	ZIP				Single				100			
Distant traps: mean count = 0.5												
	AIC	R^2^	aRMSE	rRMSE	AIC	R^2^	aRMSE	rRMSE	AIC	R^2^	aRMSE	rRMSE
D1 (a)	1.48	0.40	1.48	2.96	1.67	0.35	1.56	3.12	1.14	0.37	1.37	**2.74**
D1 (b)	1.55	0.30	1.97	3.94	1.80	0.30	1.89	3.72	1.33	0.27	1.88	3.76
D2 (a)	1.67	0.14	2.13	4.26	2.01	0.21	2.01	4.02	1.39	0.24	1.84	3.68
D2 (b)	1.72	0.08	2.18	4.36	2.13	0.16	2.16	4.32	1.47	0.16	1.97	3.94
D3 (a)	1.64	0.19	2.07	4.14	1.91	0.25	1.9	3.8	1.30	0.30	1.7	3.4
D3 (b)	1.68	0.18	2.09	4.18	1.96	0.23	2.01	4.02	1.36	0.25	2.0	4.0
Proximal traps: mean count = 10.4												
		R^2^	aRMSE	rRMSE		R^2^	aRMSE	rRMSE		R^2^	aRMSE	rRMSE
D1 (a)	11.56	0.37	26.57	2.55	13.10	0.52	26.87	2.58	11.07	0.53	26.24	**2.52**
D1 (b)	13.62	0.27	28.49	2.74	15.40	0.44	28.65	2.75	13.23	0.44	28.69	2.76
D2 (a)	14.99	0.21	29.55	2.84	17.04	0.38	29.57	2.84	14.52	0.38	29.15	2.80
D2 (b)	15.85	0.17	30.33	2.92	18.14	0.34	30.32	2.92	15.40	0.34	29.44	2.83
D3 (a)	15.26	0.17	30.15	2.90	17.29	0.37	30.11	2.90	14.59	0.38	29.49	2.84
D3 (b)	15.95	0.16	30.44	2.93	18.17	0.40	30.42	2.93	15.57	0.34	30.28	2.91

**Fig 4:**
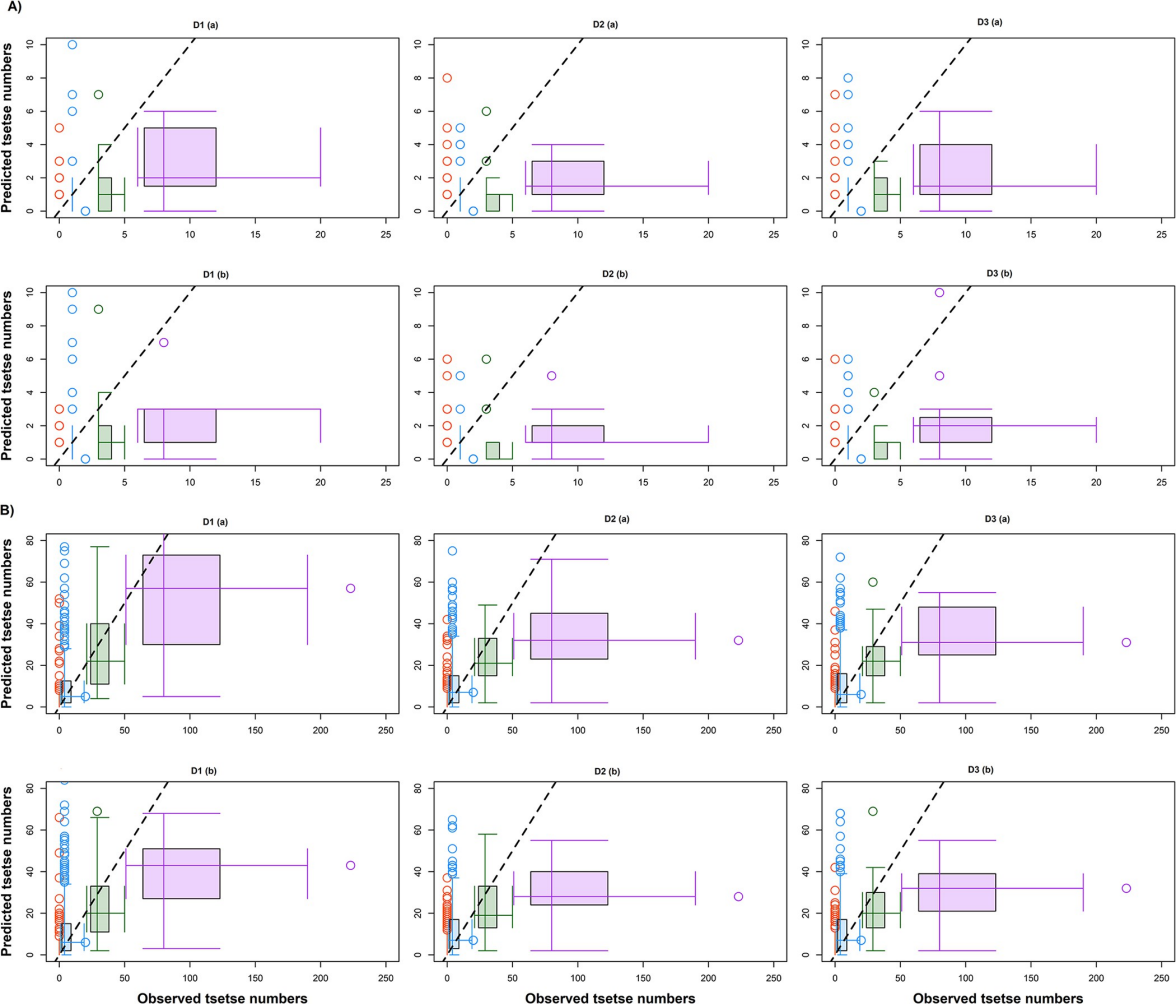
Double boxplots showing the predicted tsetse counts verses the observed tsetse count for the distant (A) and proximal (B) traps. The dashed black line is the 1:1 line where a closer alignment to the center of the box-whisker indicates a better model fit. The circles are the observations that fall outside 1.5 times the interquartile range. D1 represents 10-day time-varying variables (t1 to t6), D2 represents t1 and averaged t2-t6 variables, and D3 represents averaged t1-t3 and t4-t6 variables. The (a) next to the dataset are models with LST while (b) are those without.

As the R^2^ values were found to be low, the 1:1 scatter plot appeared visually unappealing. Consequently, to compare the predicted tsetse count with the actual count, we adopted a binning approach that was based on the observed tsetse counts rather than individual observations. We grouped the data into four bins based on observed count for distant traps (0, 1–2, 3–5, and >5 per 8-day trapping period) and for proximal traps (0, 1–20, 21–50, and >50 per 8-day trapping period) and generated double boxplots [47] across the different datasets. The double boxplots (**Fig 4**) show that the predicted values were closer to observed values (i.e., close to the one-to-one line) for low tsetse numbers, but as observed numbers increased the predicted values were lower.

### Factors influencing the temporal dynamics of tsetse numbers over time

**Fig 5:**
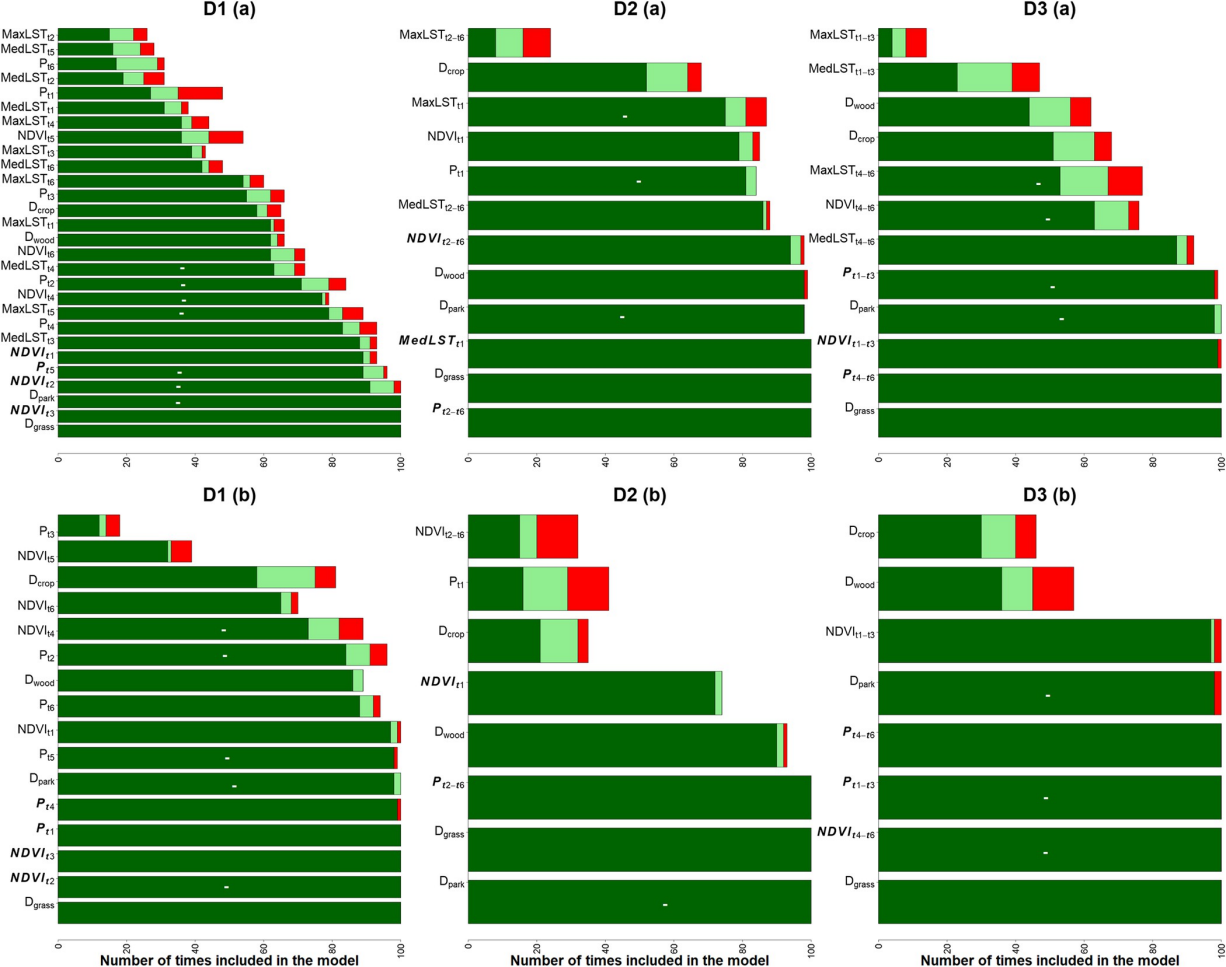
The significance of predictor variables from the randomised (100) Poisson GLM models for distant trap groups. Dark green indicates variables added with p-value < 0.05, light green indicates p-value 0.05–0.1, and red indicates p-value > 0.1. Bold italic variables are the top 4 temporal varying variables explaining tsetse numbers. Green bars with a—sign indicate variables with a negative relationship while the rest have a positive relationship. D1 includes t1-t6, D2 includes t1 and averaged t2-t6, and D3 includes averaged t1-t3 and t4-t6. The models labelled with (a) are those with LST and (b) those without LST.

Although the models had limited success in explaining tsetse number fluctuations, they offer valuable insights for deploying tsetse control strategies based on identified important variables. Models incorporating 10-day variables (D1) performed better, but highly correlated variables could obscure individual effects, making it difficult to determine important variables. Therefore, the findings discussed hereafter are based on various modelling strategies fitted using datasets D2 and D3, which were constructed with less correlated variables. The total previous precipitation (31–60 days and 1–30 days) and the succeeding NDVI values (1–30 days), were consistently identified by all modelling strategies as the most significant temporal varying variables in explaining changes in tsetse flies (**Figs 5, S1, and S2**) for distant group. Total precipitation 31–60 days (P_t4-t6_) and average NDVI 1–30 days (NDVI_t1-t30_) positively related to tsetse, while total precipitation 1–30 days (P_t1-t3_) showed a negative correlation.

**Fig 6:**
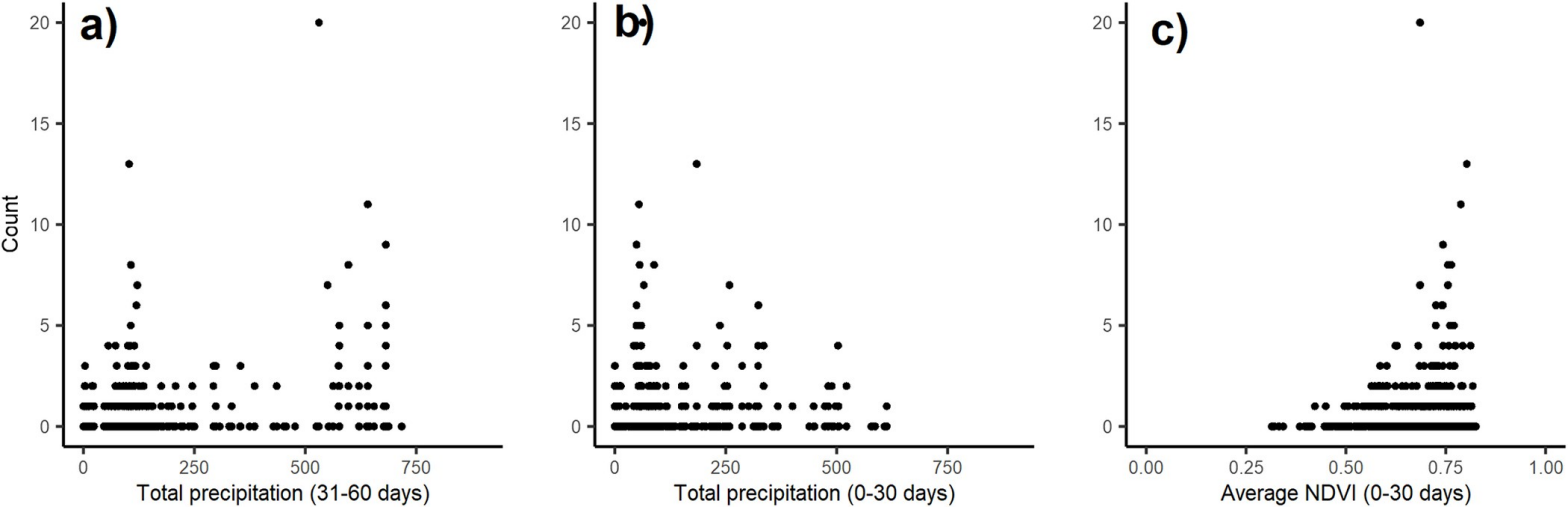
Scatterplots showing the relationship between observed tsetse numbers and the two most important variables for the distant traps: a) = P_t3-t6_; b) = P_t1-t3_ and c) = NDVI_t1-t3_.

The found relationships between tsetse numbers and NDVI or precipitation for the distant traps were to some extent visible when plotting the explanatory variables against the tsetse observation data. After a month of increased rainfall, tsetse populations tended to rise, even in areas and periods with more than 500 mm of rain (**Fig 6A**). However, if the rain persisted for longer than a month, tsetse numbers began to decline in locations and times with over 500mm of total rainfall (**Fig 6B**). As for NDVI, it was observed that high tsetse numbers occurred at times and locations where NDVI values exceeded 0.7 (**Fig 6C**).

**Fig 7:**
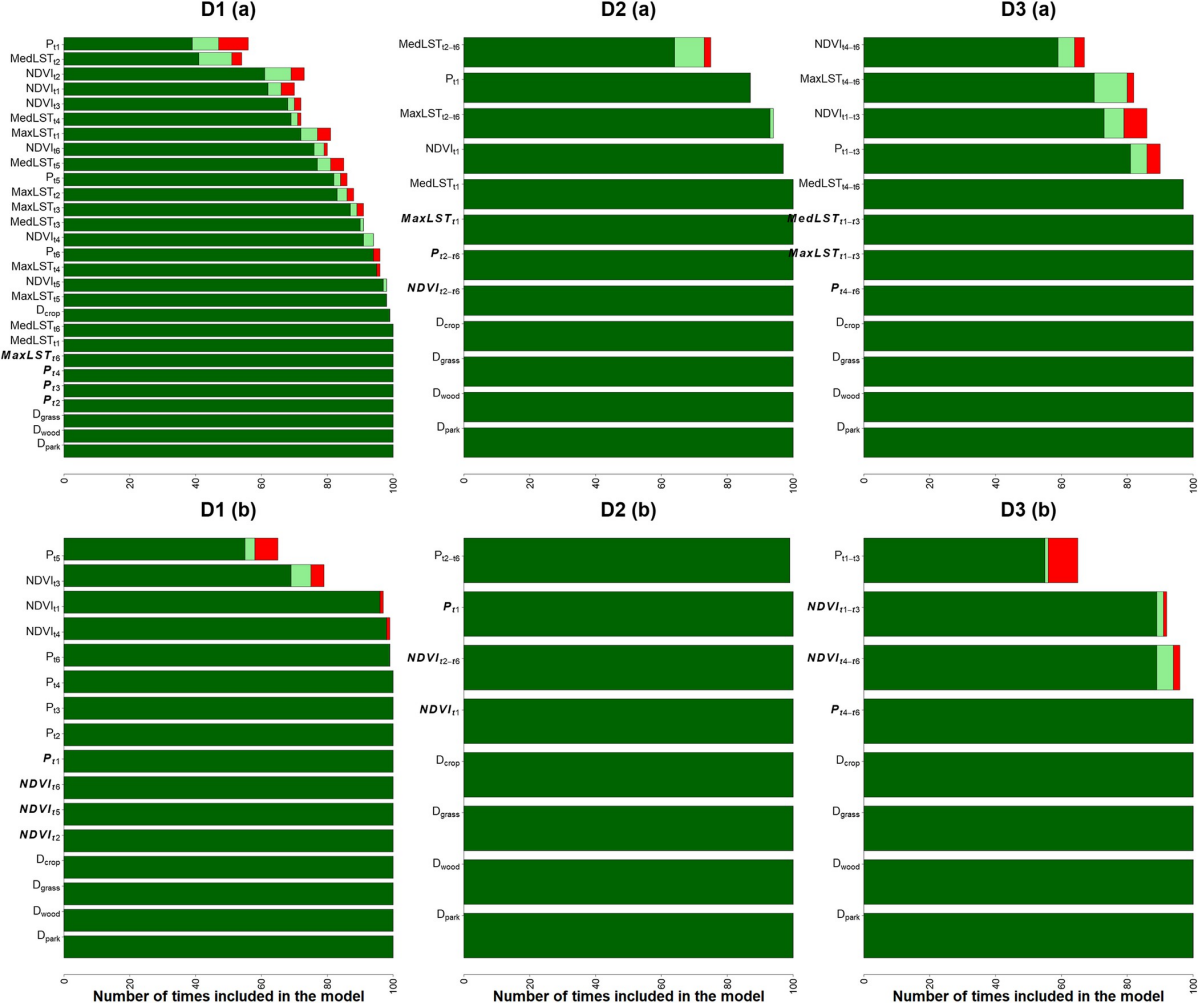
The significance of predictor variables from the randomised (100) Poisson GLM models for the proximal group. Dark green indicates variables added with p-value < 0.05, light green indicates p-value 0.05–0.1, and red indicates p-value > 0.1. Bold italic variables are the top 4 temporal varying variables explaining tsetse numbers. D1 includes t1-t6, D2 includes t1 and averaged t2-t6, and D3 includes averaged t1-t3 and t4-t6. The models labeled with (a) are those with LST and (b) those without LST.

In all the modelling strategies applied to proximal traps, almost all variables were consistently deemed important, indicating that it was difficult to differentiate which variables explained the changes in tsetse numbers near the reserve boundary (**Figs 7**, **S3,** and **S4**). However, static variables such as distances to various landcovers and the reserve boundary were repeatedly identified as significant, suggesting that they were more effective in explaining the temporal dynamics of tsetse than the temporally varying variables.

## Discussion

This study aimed to analyse the spatial and temporal abundance dynamics of *G*. *pallidipes* using trapping data and investigate potential environmental and weather factors that explain these patterns. Our findings showed that tsetse fly numbers decreased with increasing distance from the reserve boundary, with most captures occurring within a 1.0 km radius of the reserve. However, the models could not identify crucial variables that explain tsetse numbers in traps within 1.0 km of the reserve because all variables were deemed important. For traps located further away (>1.0 km), the most significant variables in explaining the temporal dynamics of tsetse numbers were total precipitation and average NDVI values. An increase in rainfall for a month resulted in increased tsetse numbers, but prolonged rainfall for more than a month led to a decline. For NDVI, tsetse numbers increased with increasing NDVI values a month after the onset of increased rains.

While rainfall may not have a direct impact on certain aspects of tsetse activities, such as larval development or production rate, it can indirectly affect the dynamics of tsetse populations. For example, rainfall often leads to an increase in vegetation cover, which is crucial for tsetse breeding and resting [12,24]. This might explain why tsetse numbers in distant traps rose in tandem with increasing NDVI values shortly after the start of the rainy season. The amount of rainfall is also a significant determinant of the spatial and temporal variability of soil moisture [48]. Moist soil provides an ideal location for the deposition of larvae, making it essential for their survival [24,36,49]. In instances where rainfall is light and soil can quickly absorb the water, the soil moisture content increases rapidly, providing a suitable environment for deposited pupae. However, heavy rainfall or already saturated soil can result in surface run-off or flooding, which may lead to the submersion and death of burrowed pupae [17,50–52]. These two scenarios could explain the increase in tsetse fly populations at the onset of the rainy season, followed by a decline as the rains persisted. Additionally, studies conducted in various locations have reported higher numbers of tsetse flies caught during the dry season than the wet season [17,20], which could be due to pupae loss because of flooding.

Ambient temperature plays an important role in shaping the temporal dynamics of tsetse flies, as it influences their physiological processes [12,13,53]. The mortality rates of adult tsetse increase as temperatures rise [13,14,54], while the overall survival rate of tsetse decreases at higher temperatures [14,55,56] lowering tsetse numbers. Also, elevated temperatures enhance larval production rates and accelerate the emergence of pupae [13], potentially resulting in a greater number of tsetse. When the temperature drops below a certain threshold, adult tsetse flies may experience chill coma [15], female flies’ ovaries may develop eggs at a slower rate [17], and pupae may take longer to emerge [16,17], resulting in reduced tsetse populations. Although we utilized LST as a proxy for ambient temperature in our study, it was not found to be a significant factor. However, Lin *et al*. [15] highlighted that caution should be taken when using LST as a substitute for ambient temperature since daytime air temperature has been reported to be lower than the estimated LST due to the high influence of small variations in heat fluxes.

Although the explanatory variables used in our models had some predictive power, prediction of the fluctuations in tsetse populations over time could not be generated with a high percentage of explained model variance. Given the different modelling approaches tested, this suggests that the three time-varying predictors used—NDVI, LST, and precipitation—could insufficiently represent the dynamics of tsetse fly populations. For instance, LST cannot accurately represent the ambient temperature [15], which is thought to be more important for tsetse flies [13,38] and this could have impacted the accuracy of our models. Future studies can obtain finer estimates of ambient temperature by building predictive models that can be fitted with LST and elevation as predictor variables [15]. Alternatively, data from several weather stations in the region can be combined with relevant satellite-based information to predict ambient temperature over large areas.

Tsetse flies need adequate blood meals to breed, and therefore, the presence and abundance of host animals is crucial in determining tsetse fly populations. Ngonyoka et al [57] found a positive correlation between tsetse species (including *G*. *pallidipes*) and animal host abundance in Maasai Steppe and they suggest that animal host information can explain seasonal changes in tsetse fly population. Notably, the availability of information on blood-hosts for tsetse to feed on was not included in our study. Therefore, in future studies, it would be beneficial to integrate host availability data. To collect host data, one can observe their relative abundance and activities, including tracks, droppings, burrows, and shelters during trap visits [57]. Alternatively, tracking livestock and wildlife using Global Positioning Satellite (GPS) tags, camera traps, or GPS fixes from herders could be useful.

Despite the low model performances, important factors were identified for traps beyond 1.0 km of the reserve boundary, providing information for targeted tsetse control strategies around Shimba Hills National Reserve. Our results suggest deploying tsetse control within one month of increased rain in areas with NDVI values greater than 0.7 in these localities. Previous studies suggest using insecticide-treated traps, targets, and livestock to increase adult mortality rates [58–61]. Continuous management within 1.0 km of the reserve boundary is necessary due to high tsetse numbers. The KWS prohibits tsetse management within the reserve since their primary mandate is to protect wild animals whether good or bad. A potential strategy for managing tsetse in the surrounding areas is to install insecticide-treated targets or traps at regular intervals along the perimeter, creating a tsetse barrier. The employment of an odour-baited target for minimizing the tsetse population and thwarting re-infestation has proven effective in Zimbabwe [62,63]. Similarly, this strategy has demonstrated success in reducing tsetse populations in the Shimba Hills National Reserve, but it necessitates consistent funding to be sustainable.

## Conclusion

Tsetse fly abundance varied greatly across space and time. Beyond 1.0 km of the reserve boundary, our findings indicate that increases in rainfall one month prior to sampling, as well as subsequent NDVI values, increased the likelihood of high abundance. As such, spatial-temporal information on rainfall and NDVI can help to assess when to expect seasonal increases in tsetse abundance and the timing and location of control efforts. This information can be used as a decision-support tool for improved and effective intervention strategies.

## Supporting information

S1 Fig Slope coefficient plots for the single GLM models for the distant traps. D1 (a 10-day variable), D2 (t1 and the averaged t2-t6), or D3 (the averaged t1-t3 and t4-t6). (a) and (b) next to the various dataset is models that included LST and those that did not. The red values represent variables with a negative relationship, while the blue values indicate a positive relationship. Significant relationships are denoted by an asterisk symbol. The variables av1, av2, and av3 correspond to the averaged t2-t6, averaged t1-t3, and averaged t4-t6, respectively.
(TIF)

S2 Fig Slope coefficient plots for the ZIP models for the distant traps. D1 = 10-day variable, D2 = t1 and averaged t2-t6, D3 = averaged t1-t3 and t4-t6. (a) and (b) next to the various dataset is models that included LST and those that did not. The red color value shows variables with negative relationship while the blue values show a positive relationship. The * symbol indicate the level of significance. av1 = averaged t2-t6; av2 = averaged t1-t3 and av3 = averaged t4-t6.
(TIF)

S3 Fig Slope coefficient plots for the single GLM models for the proximal traps. D1 = 10-day variable, D2 = t1 and averaged t2-t6, D3 = averaged t1-t3 and t4-t6. (a) and (b) next to the various dataset is models that included LST and those that did not. The red color value shows variables with negative relationship while the blue values show a positive relationship. The * symbol indicate the level of significance. av1 = averaged t2-t6; av2 = averaged t1-t3 and av3 = averaged t4-t6.
(TIF)

S4 Fig Slope coefficient plots for the ZIP models for the proximal traps. D1 = 10-day variable, D2 = t1 and averaged t2-t6, D3 = averaged t1-t3 and t4-t6. (a) and (b) next to the various dataset is models that included LST and those that did not. The red color value shows variables with negative relationship while the blue values show a positive relationship. The * symbol indicate the level of significance. av1 = averaged t2-t6; av2 = averaged t1-t3 and av3 = averaged t4-t6.
(TIF)

## References

[1] Dargie J. Tsetse and trypanosomiasis information. Rome, Italy: FAO; 2015. 118p.

[2] Wint W. Tsetse fly distribution data [Internet]. Environmental Research Group Oxford [Data CD prepared by Environmental Research Group Oxford Ltd for the Insect Pest Control Section, Joint FAO/IAEA Division of Nuclear Techniques in Food and Agriculture, International Atomic Energy Agency]. 2003.

[3] WHO. Benin, Uganda and Rwanda eliminate human African trypanosomiasis as a public health problem [Internet]. World Health Organization. 2022 [cited 2023 Apr 23]. Available from: https://www.who.int/news/item/24-05-2022-benin—uganda-and-rwanda-eliminate-human-african-trypanosomiasis-as-a-public-health-problem.

[4] Diall O, Cecchi G, Wanda G, Argilés-Herrero R, Vreysen MJB, Cattoli G, et al. Developing a Progressive Control Pathway for African Animal Trypanosomosis. Trends Parasitol. 2017 Jul;33(7):499–509. doi: 10.1016/j.pt.2017.02.005 28456474

[5] Giordani F, Morrison LJ, Rowan TG, De Koning HP, Barrett MP. The animal trypanosomiases and their chemotherapy: a review. Parasitology. 2016;143(14):1862–89. doi: 10.1017/S0031182016001268 27719692 PMC5142301

[6] Assefa S, Shibeshi W. Drug resistance in African animal trypanosomes. African J Microbiol Res. 2018;12(17):380–6.

[7] Chitanga S, Marcotty T, Namangala B, van den Bossche P, van den Abbeele J, Delespaux V. High prevalence of drug resistance in animal trypanosomes without a history of drug exposure. PLoS Negl Trop Dis. 2011 Dec;5(12). doi: 10.1371/journal.pntd.0001454 22206039 PMC3243716

[8] Degneh E, Ashenafi H, Kassa T, Kebede N, Shibeshi W, Asres K, et al. Trypanocidal drug resistance: A threat to animal health and production in Gidami district of Kellem Wollega Zone, Oromia Regional State, Western Ethiopia. Prev Vet Med. 2019 Jul;168:103–7. doi: 10.1016/j.prevetmed.2019.03.017 31076189

[9] Geerts S, Holmes PH, Eisler MC, Diall O. African bovine trypanosomiasis: the problem of drug resistance. Trends Parasitol. 2001 Jan;17(1):25–8. doi: 10.1016/s1471-4922(00)01827-4 11137737

[10] Holmes P. Tsetse-transmitted trypanosomes—Their biology, disease impact and control. J Invertebr Pathol. 2013 Mar; p. S11–4. doi: 10.1016/j.jip.2012.07.014 22841638

[11] Cattand P, Desjeux P, Guzmán MG, Jannin J, Kroeger A, Medici A, et al. Tropical Diseases Lacking Adequate Control Measures: Dengue, Leishmaniasis, and African Trypanosomiasis. Dis Control Priorities Dev Ctries. The International Bank for Reconstruction and Development / The World Bank; 2006. 21250331

[12] Lord JS, Hargrove JW, Torr SJ, Vale GA. Climate change and African trypanosomiasis vector populations in Zimbabwe’s Zambezi Valley: A mathematical modelling study. Thomson M, editor. PLOS Med. 2018 Oct;15(10):e1002675. doi: 10.1371/journal.pmed.1002675 30346952 PMC6197628

[13] Are EB, Hargrove JW. Extinction probabilities as a function of temperature for populations of tsetse (Glossina spp.). PLoS Negl Trop Dis. 2020 May;14(5):e0007769. doi: 10.1371/journal.pntd.0007769 32379749 PMC7237048

[14] Terblanche JS, Clusella-Trullas S, Deere JA, Chown SL. Thermal tolerance in a south-east African population of the tsetse fly Glossina pallidipes (Diptera, Glossinidae): Implications for forecasting climate change impacts. J Insect Physiol. 2008 Jan;54(1):114–27. doi: 10.1016/j.jinsphys.2007.08.007 17889900

[15] Lin S, Moore NJ, Messina JP, DeVisser MH, Wu J. Evaluation of estimating daily maximum and minimum air temperature with MODIS data in east Africa. Int J Appl Earth Obs Geoinf. 2012 Aug;18(1):128–40.

[16] Harley JMB. The influence of temperature on reproduction and development in four species of Glossina (Diptera: Muscidae). Proc R Entomol Soc London Ser A, Gen Entomol. 1968 Dec;43(10–12):170–7.

[17] Lukaw YS, Abdelrahman MM, Mohammed YO, Ochi EB, Elrayah IE. Factors influencing seasonal abundance of Glossina fuscipes fuscipes (*Glossina*: Glossinidae) in Kajo-Keji County, South Sudan. Curr Res J Biol Sci. 2014 Nov;6(6):222–8.

[18] Nnko HJ, Gwakisa PS, Ngonyoka A, Sindato C, Estes AB. Potential impacts of climate change on geographical distribution of three primary vectors of African Trypanosomiasis in Tanzania’s Maasai Steppe: G. m. morsitans, G. pallidipes and G. swynnertoni. PLoS Negl Trop Dis. 2021 Feb;15(2):e0009081. doi: 10.1371/journal.pntd.0009081 33571190 PMC7904224

[19] Omoogun GA, Dipeolu OO, Akinboade OA. Distribution and seasonal variation of tsetse population in the egbe area of kwara state, Nigeria. Int J Trop Insect Sci. 1989 Oct;10(05):713–8.

[20] Ngonyoka A, Gwakisa PS, Estes AB, Salekwa LP, Nnko HJ, Hudson PJ, et al. Patterns of tsetse abundance and trypanosome infection rates among habitats of surveyed villages in Maasai steppe of northern Tanzania. Infect Dis Poverty. 2017 Dec;6(1):126. doi: 10.1186/s40249-017-0340-0 28866983 PMC5582388

[21] Rouse JW. J, Haas RH, Schell JA, Deering DW, Haas RH, Schell JA, et al. monitoring vegetation systems in the great plains with erts. NASA Goddard Sp Flight Cent 3d ERTS-1 Symp, Vol 1, Sect A. 1974;

[22] Pettorelli N, Ryan S, Mueller T, Bunnefeld N, Jedrzejewska B, Lima M, et al. The Normalized Difference Vegetation Index (NDVI): unforeseen successes in animal ecology. ClRes. 2011;46(1):15–27.

[23] Rogers DJ. Satellite imagery, tsetse and trypanosomiasis in Africa. Prev Vet Med. 1991 Dec;11(3–4):201–20.

[24] Gachoki S, Groen T, Vrieling A, Okal M, Skidmore A, Masiga D. Satellite-based modelling of potential tsetse (Glossina pallidipes) breeding and foraging sites using teneral and non-teneral fly occurrence data. Parasites Vectors 2021 141. 2021 Sep;14(1):1–18. doi: 10.1186/s13071-021-05017-5 34583766 PMC8479894

[25] DeVisser MH, Messina JP. Optimum land cover products for use in a Glossina-morsitans habitat model of Kenya. Int J Health Geogr. 2009 Jun;8(1):1–20. 19563674 10.1186/1476-072X-8-39 PMC2710327

[26] Robinson T, Rogers D, Williams B. Univariate analysis of tsetse habitat in the common fly belt of Southern Africa using climate and remotely sensed vegetation data. Med Vet Entomol. 1997 Jul;11(3):223–34. doi: 10.1111/j.1365-2915.1997.tb00400.x 9330253

[27] Saini RK, Orindi BO, Mbahin N, Andoke JA, Muasa PN, Mbuvi DM, et al. Protecting cows in small holder farms in East Africa from tsetse flies by mimicking the odor profile of a non-host bovid. Solano P, editor. PLoS Negl Trop Dis. 2017 Oct;11(10):e0005977. doi: 10.1371/journal.pntd.0005977 29040267 PMC5659797

[28] Ebhodaghe FI, Bastos ADS, Okal MN, Masiga DK. Entomological assessment of tsetse-borne trypanosome risk in the Shimba Hills human-wildlife-livestock interface, Kenya. Front Vet Sci. 2022 Aug;9:1124. doi: 10.3389/fvets.2022.931078 36051538 PMC9424651

[29] Lin S, DeVisser MH, Messina JP. An agent-based model to simulate tsetse fly distribution and control techniques: a case study in Nguruman, Kenya. Ecol Modell. 2015 Oct;314:80–9. doi: 10.1016/j.ecolmodel.2015.07.015 26309347 PMC4545571

[30] Glover PE. The importance of ecological studies in the control of tsetse flies. Bull World Health Organ. 1967;37(4):581–614. 4874781 PMC2554373

[31] Ebhodaghe FI, Okal MN, Kalayou S, Bastos ADS, Masiga DK, Kalayou MN;, et al. Tsetse Bloodmeal Analyses Incriminate the Common Warthog Phacochoerus africanus as an Important Cryptic Host of Animal Trypanosomes in Smallholder Cattle Farming Communities in Shimba Hills, Kenya. Pathog 2021, Vol 10, Page 1501. 2021 Nov;10(11):1501. doi: 10.3390/pathogens10111501 34832656 PMC8623152

[32] Kulohoma BW, Wamwenje SAO, Wangwe II, Masila N, Mirieri CK, Wambua L. Prevalence of trypanosomes associated with drug resistance in Shimba Hills, Kwale County, Kenya. BMC Res Notes. 2020 Apr;13(1):234. doi: 10.1186/s13104-020-05077-3 32349785 PMC7191804

[33] Muriithi BW, Gathogo NG, Diiro GM, Kidoido MM, Okal MN, Masiga DK. Farmer perceptions and willingness to pay for novel livestock pest control technologies: A case of tsetse repellent collar in Kwale County in Kenya. PLoS Negl Trop Dis. 2021 Aug;15(8):e0009663. doi: 10.1371/journal.pntd.0009663 34403426 PMC8396722

[34] Isherwood F, Duffy B., Glasgow J., Lee-Jones F, Weitz B. Further studies of the food of tsetse flies. J Anim Ecol. 1961;

[35] Hargrove J. Tsetse population dynamics. Trypanos. 2004;113–37.

[36] Lambrecht FL. Aspects of evolution and ecology of tsetse flies and trypanosomiasis in prehistoric African environment. J Afr Hist. 1964;5(1):1–24.

[37] Nash TAM. A Statistical Analysis of the Climatic Factors Influencing the Density of Tsetse Flies, Glossina morsitans Westw. J Anim Ecol. 1933 Nov;2(2):197.

[38] Hargrove JW. Extinction probabilities and times to extinction for populations of tsetse flies *Glossina spp*. (Diptera: Glossinidae) subjected to various control measures. Bull Entomol Res. 2005 Feb;95(1):13–21. 15705210 10.1079/ber2004335

[39] Phelps RJ, Clarke GPY. Seasonal elimination of some size classes in males of Glossina morsitans morsitans Westw. (Diptera, Glossinidae). Bull Entomol Res. 1974;64(2):313–24.

[40] Lyashevska O, Brus DJ, van der Meer J. Mapping species abundance by a spatial zero-inflated Poisson model: a case study in the Wadden Sea, the Netherlands. Ecol Evol. 2016 Jan;6(2):532–43. doi: 10.1002/ece3.1880 26843936 PMC4729254

[41] Zeileis A, Kleiber C, Jackman S. Regression Models for Count Data in R. 2008;

[42] Udokang Anietie MA, Raji Surajudeen E, Bello Latifat Kemi T. An Empirical Study of Generalized Linear Model for Count Data. J Appl Comput Math. 2015;04(05).

[43] Jackman S, Tahk A, Zeileis A, Maimone C, Fearon J, Maintainer ZM. Political Science Computational Laboratory. 2020;

[44] Zeileis A, Jackman S, Rip-Ley B, Maintainer PB, Wang Z. Regularized Linear Models (’mpath’). 2022;

[45] Ripley B. Package “MASS.” 2022;

[46] Bautista-Cespedes O V., Willemen L, Castro-Nunez A, Groen TA. The effects of armed conflict on forest cover changes across temporal and spatial scales in the Colombian Amazon. Reg Environ Chang. 2021 Sep;21(3):1–16.

[47] Tomizonor. Draw a Double Box Plot Chart (2-Axes Box Plot; Box Plot Correlation Diagram) in R [Internet]. 2013 [cited 2023 Apr 20]. Available from: https://tomizonor.wordpress.com/2013/03/15/double-box-plot/.

[48] Sehler R, Li J, Reager J, Ye H. Investigating Relationship Between Soil Moisture and Precipitation Globally Using Remote Sensing Observations. J Contemp Water Res Educ. 2019 Dec;168(1):106–18.

[49] Buxton PA. The natural history of tsetse flies. Geogr J. 1956 Mar;122(1):115.

[50] Signaboubo D, Payne VK, Moussa IMA, Hassane HM, Berger P, Kelm S, et al. Diversity of tsetse flies and trypanosome species circulating in the area of Lake Iro in southeastern Chad. Parasites and Vectors. 2021 Dec;14(1):1–11. 34078431 10.1186/s13071-021-04782-7 PMC8173974

[51] Muzari MO, Hargrove JW. Artificial larviposition sites for field collections of the puparia of tsetse flies Glossina pallidipes and G. m. morsitans (Diptera: Glossinidae). Bull Entomol Res. 2005 Jun;95(3):221–9. doi: 10.1079/ber2004354 15960876

[52] Hargrove JW, Muzari MO. Artificial Warthog Burrows Used to Sample Adult and Immature Tsetse (Glossina spp) in the Zambezi Valley of Zimbabwe. PLoS Negl Trop Dis. 2015 Mar;9(3). doi: 10.1371/journal.pntd.0003565 25786253 PMC4364979

[53] Moore S, Shrestha S, Tomlinson KW, Vuong H. Predicting the effect of climate change on African trypanosomiasis: integrating epidemiology with parasite and vector biology. J R Soc Interface. 2012 May;9(70):817. doi: 10.1098/rsif.2011.0654 22072451 PMC3306657

[54] Moore N, Messina J. A Landscape and Climate Data Logistic Model of Tsetse Distribution in Kenya. Jones JH, editor. PLoS One. 2010 Jul;5(7):e11809. doi: 10.1371/journal.pone.0011809 20676406 PMC2910741

[55] Pagabeleguem S, Ravel S, Dicko AH, Vreysen MJB, Parker A, Takac P, et al. Influence of temperature and relative humidity on survival and fecundity of three tsetse strains. Parasites and Vectors. 2016 Sep;9(1):1–13. doi: 10.1186/s13071-016-1805-x 27682638 PMC5041576

[56] Buxton PA, Lewis D. Climate and tsetse flies: laboratory studies upon Glossina submorsitans and tachinoides. Philos Trans R Soc Lond B Biol Sci. 1934 Dec;224(512):175–240.

[57] Ngonyoka A, Gwakisa PS, Estes AB, Nnko HJ, Hudson PJ, Cattadori IM. Variation of tsetse fly abundance in relation to habitat and host presence in the Maasai Steppe, Tanzania. J Vector Ecol. 2017 Jun;42(1):34–43. doi: 10.1111/jvec.12237 28504430

[58] Barclay HJ, Vreysen MJB. A dynamic population model for tsetse (Diptera: Glossinidae) area-wide integrated pest management. Popul Ecol. 2011 Jan;53(1):89–110.

[59] Percoma L, Sow A, Pagabeleguem S, Dicko AH, Serdebéogo O, Ouédraogo M, et al. Impact of an integrated control campaign on tsetse populations in Burkina Faso. Parasites and Vectors. 2018 Apr;11(1):1–13. 29703229 10.1186/s13071-017-2609-3 PMC5923030

[60] Dicko AH, Lancelot R, Seck MT, Guerrini L, Sall B, Lo M, et al. Using species distribution models to optimize vector control in the framework of the tsetse eradication campaign in Senegal. Proc Natl Acad Sci U S A. 2014 Jul;111(28):10149–54. doi: 10.1073/pnas.1407773111 24982143 PMC4104868

[61] Dransfield RD, Brightwell R, Kyorku C, Williams B. Control of tsetse fly (Diptera: Glossinidae) populations using traps at Nguruman, south-west Kenya. Bull Entomol Res. 1990;80(3):265–76.

[62] Vale GA, Lovemore DF, Flint S, Cockbill GF. Odour-baited targets to control tsetse flies, Glossina spp. (Diptera: Glossinidae), in Zimbabwe. Bull Entomol Res. 1988;78(1):31–49.

[63] Muzari MO. Odour-baited targets as invasion barriers for tsetse flies (Diptera: Glossinidae): a field trial in Zimbabwe. Bull Entomol Res. 1999;89(1):73–7.

