# Supplementary figures and images for: Evidence-based advice on timing and location of tsetse control measures in Shimba Hills National reserve, Kenya
Source: PLoS Negl Trop Dis. 2023 Jun 5;17(6):e0011398. doi: 10.1371/journal.pntd.0011398 (PMC10270600; doi:10.1371/journal.pntd.0011398)

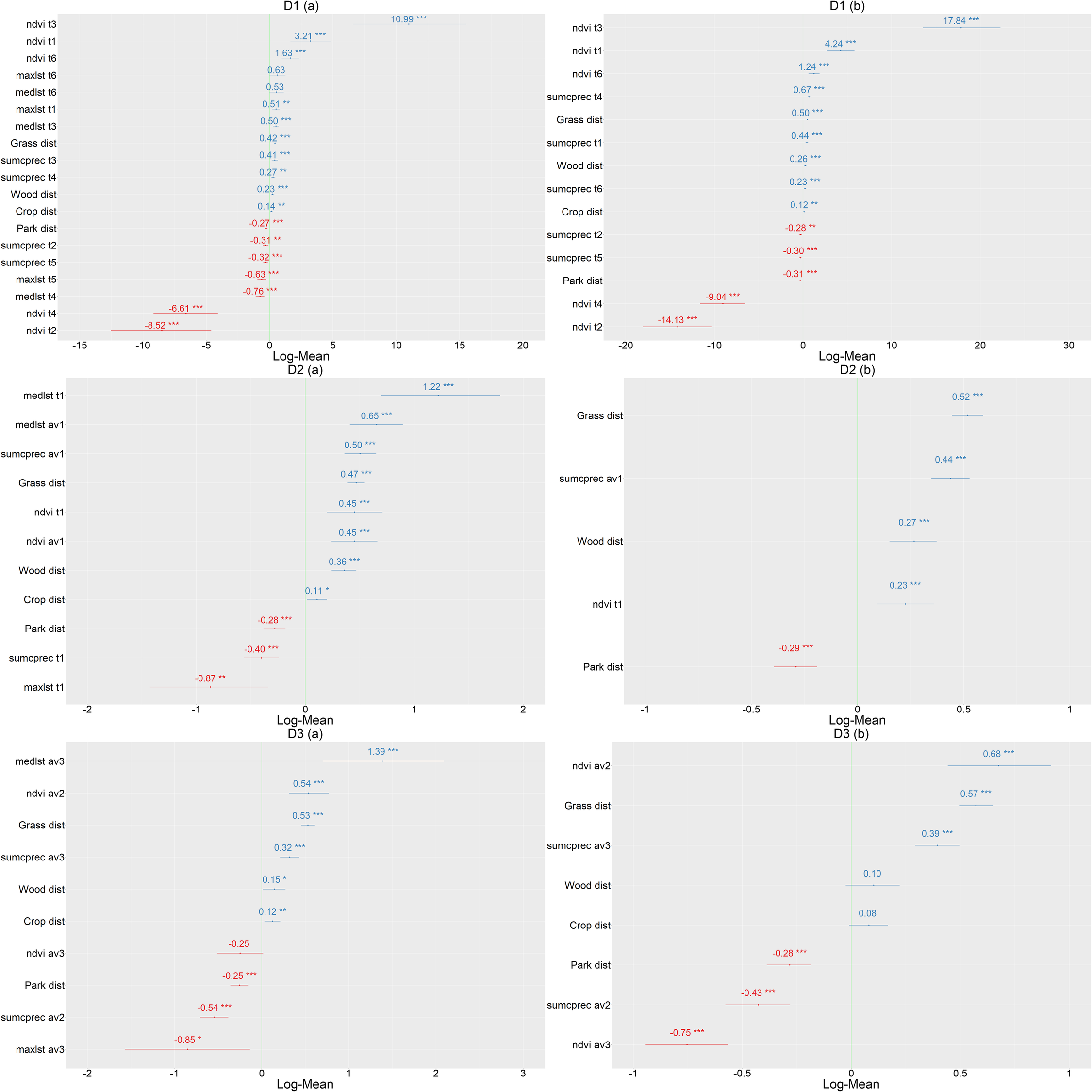

Supplement: S1 Fig — D1 (a 10-day variable), D2 (t1 and the averaged t2-t6), or D3 (the averaged t1-t3 and t4-t6). (a) and (b) next to the various dataset is models that included LST and those that did not. The red values represent variables with a negative relationship, while the blue values indicate a positive relationship. Significant relationships are denoted by an asterisk symbol. The variables av1, av2, and av3 correspond to the averaged t2-t6, averaged t1-t3, and averaged t4-t6, respectively. (TIF) [file pntd.0011398.s001.tif]

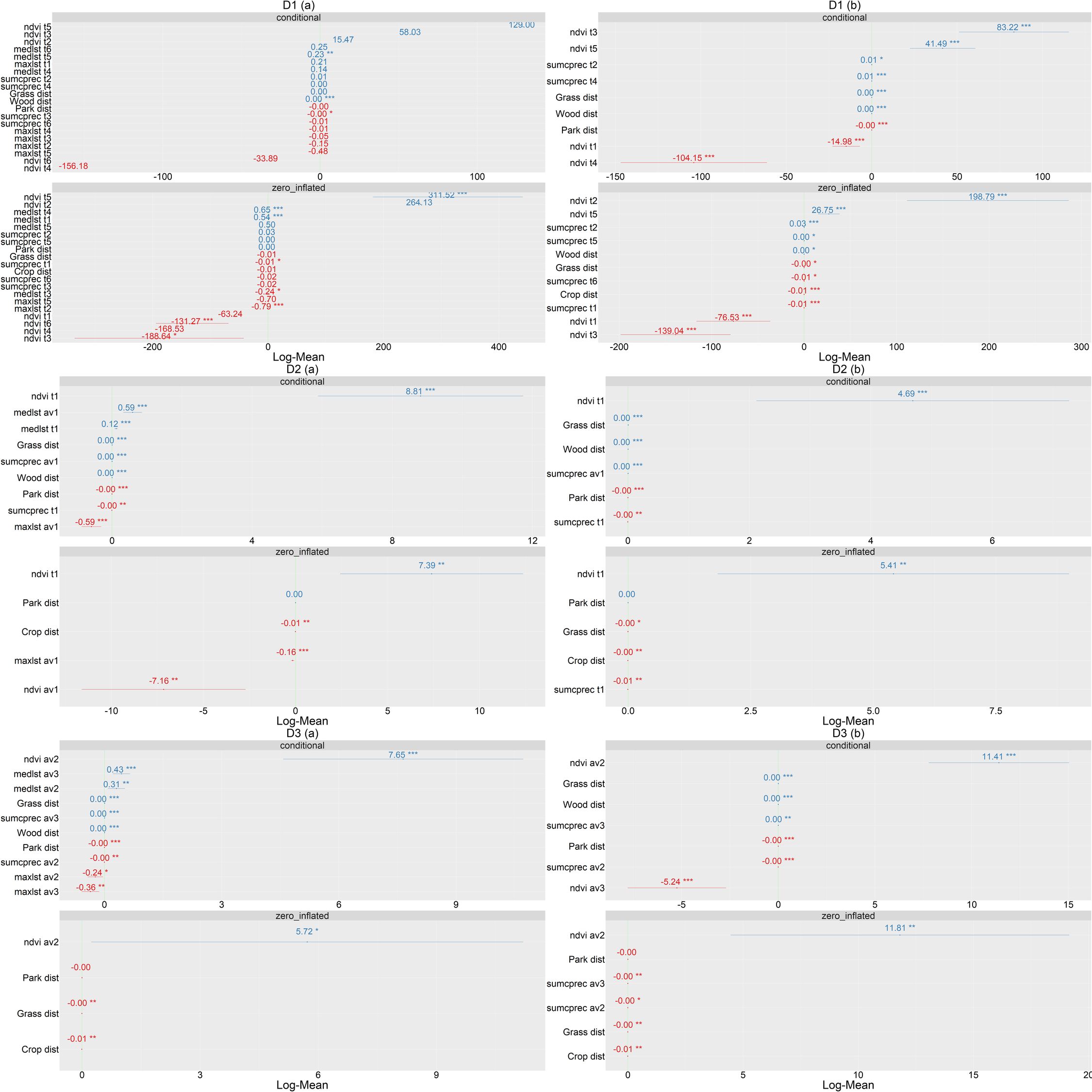

Supplement: S2 Fig — D1 = 10-day variable, D2 = t1 and averaged t2-t6, D3 = averaged t1-t3 and t4-t6. (a) and (b) next to the various dataset is models that included LST and those that did not. The red color value shows variables with negative relationship while the blue values show a positive relationship. The * symbol indicate the level of significance. av1 = averaged t2-t6; av2 = averaged t1-t3 and av3 = averaged t4-t6. (TIF) [file pntd.0011398.s002.tif]

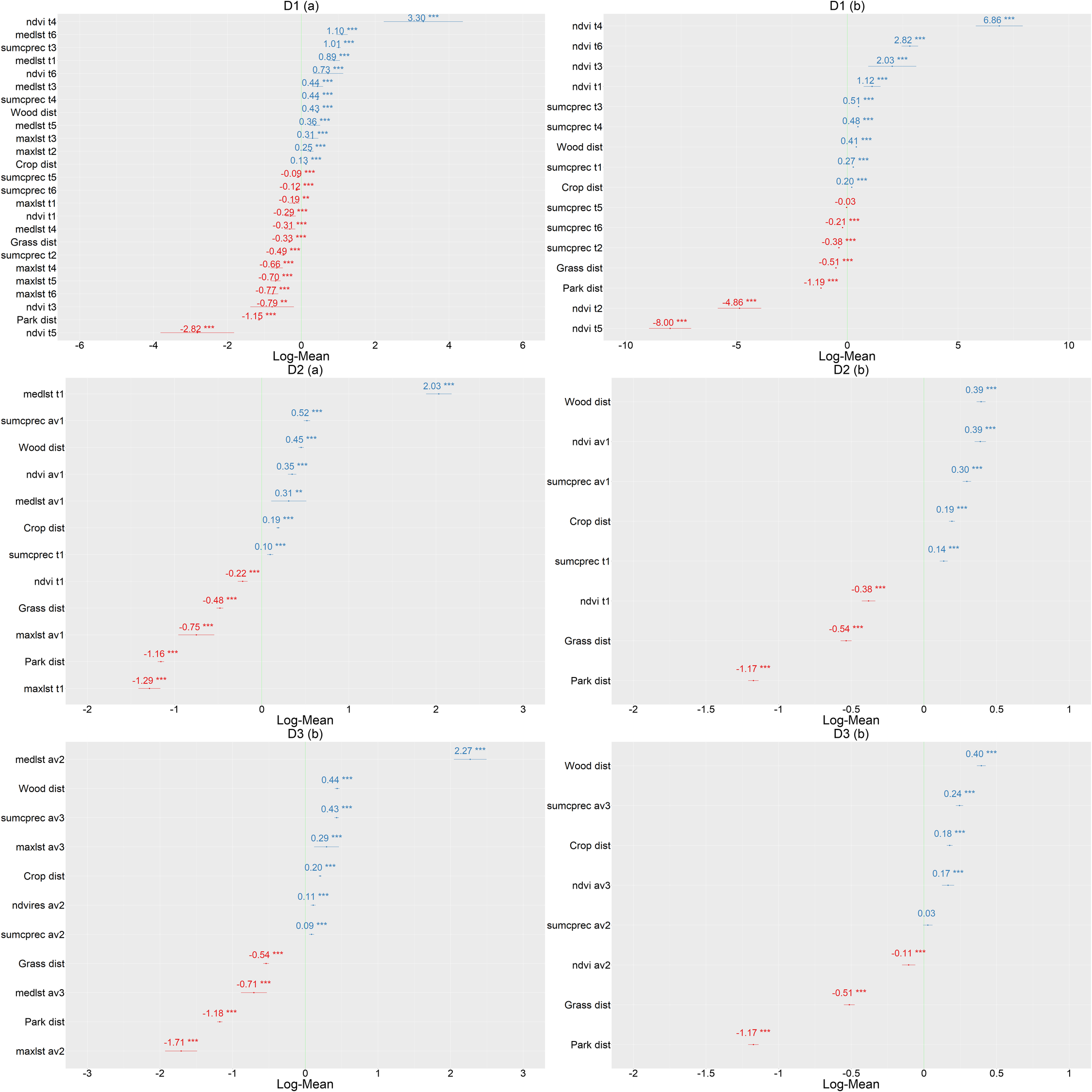

Supplement: S3 Fig — D1 = 10-day variable, D2 = t1 and averaged t2-t6, D3 = averaged t1-t3 and t4-t6. (a) and (b) next to the various dataset is models that included LST and those that did not. The red color value shows variables with negative relationship while the blue values show a positive relationship. The * symbol indicate the level of significance. av1 = averaged t2-t6; av2 = averaged t1-t3 and av3 = averaged t4-t6. (TIF) [file pntd.0011398.s003.tif]

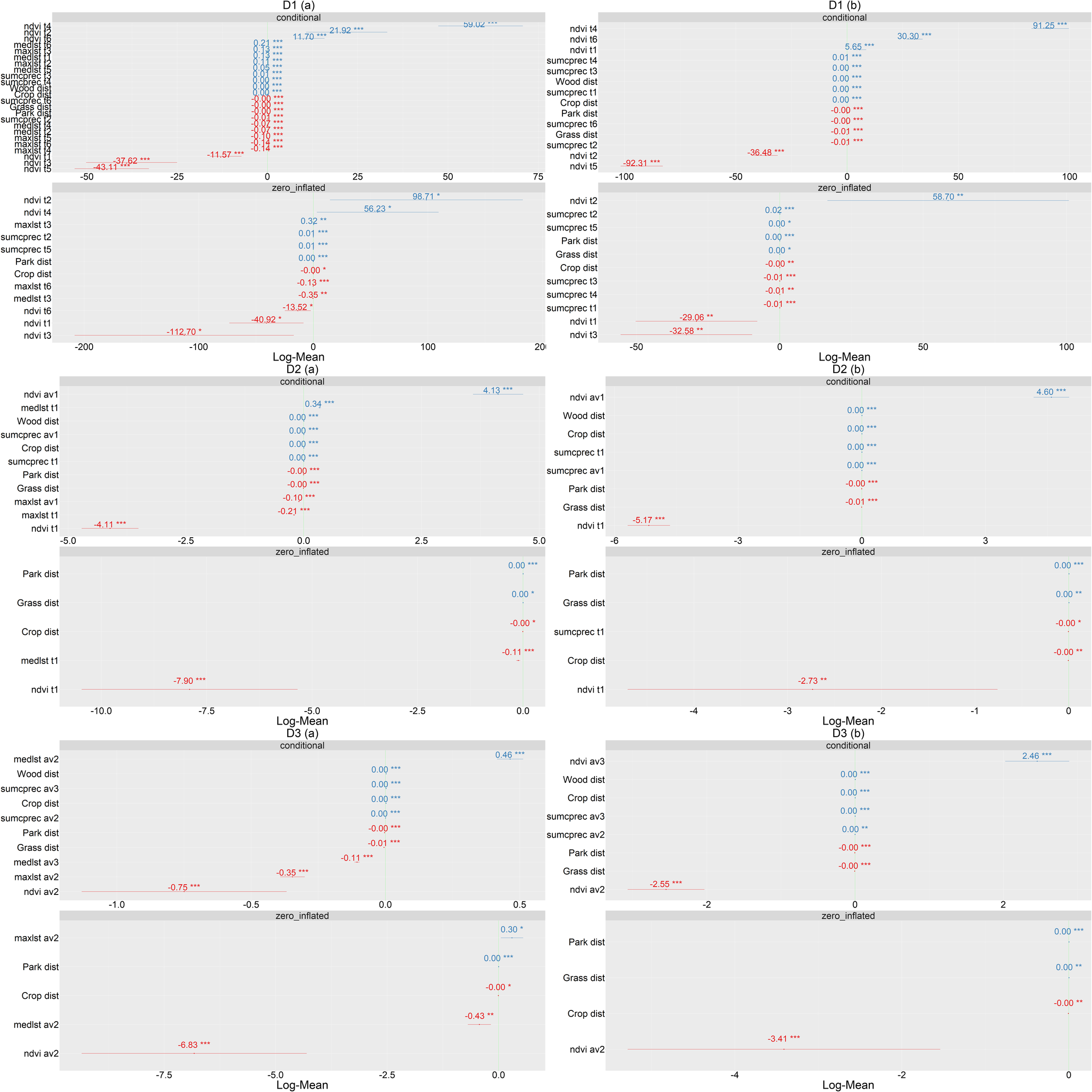

Supplement: S4 Fig — D1 = 10-day variable, D2 = t1 and averaged t2-t6, D3 = averaged t1-t3 and t4-t6. (a) and (b) next to the various dataset is models that included LST and those that did not. The red color value shows variables with negative relationship while the blue values show a positive relationship. The * symbol indicate the level of significance. av1 = averaged t2-t6; av2 = averaged t1-t3 and av3 = averaged t4-t6. (TIF) [file pntd.0011398.s004.tif]
